# Model morphing and sequence assignment after molecular replacement

**DOI:** 10.1107/S0907444913017770

**Published:** 2013-10-18

**Authors:** Thomas C. Terwilliger, Randy J. Read, Paul D. Adams, Axel T. Brunger, Pavel V. Afonine, Li-Wei Hung

**Affiliations:** a Bioscience Division, Los Alamos National Laboratory, Mail Stop M888, Los Alamos, NM 87545, USA; b Department of Haematology, University of Cambridge, Cambridge Institute for Medical Research, Cambridge CB2 0XY, England; c Physical Biosciences Division, Lawrence Berkeley National Laboratory, One Cyclotron Road, Bldg 64R0121, Berkeley, CA 94720, USA; d Departments of Molecular and Cellular Physiology, Neurology and Neurological Science, Structural Biology, Photon Science, and Howard Hughes Medical Institute, Stanford University, 318 Campus Drive West, Stanford, CA 94305, USA; e Lawrence Berkeley National Laboratory, One Cyclotron Road, Bldg 64R0121, Berkeley, CA 94720, USA

**Keywords:** morphing, model building, sequence assignment, model–map correlation, loop-building

## Abstract

A procedure termed ‘morphing’ for improving a model after it has been placed in the crystallographic cell by molecular replacement has recently been developed. Morphing consists of applying a smooth deformation to a model to make it match an electron-density map more closely. Morphing does not change the identities of the residues in the chain, only their coordinates. Consequently, if the true structure differs from the working model by containing different residues, these differences cannot be corrected by morphing. Here, a procedure that helps to address this limitation is described. The goal of the procedure is to obtain a relatively complete model that has accurate main-chain atomic positions and residues that are correctly assigned to the sequence. Residues in a morphed model that do not match the electron-density map are removed. Each segment of the resulting trimmed morphed model is then assigned to the sequence of the molecule using information about the connectivity of the chains from the working model and from connections that can be identified from the electron-density map. The procedure was tested by application to a recently determined structure at a resolution of 3.2 Å and was found to increase the number of correctly identified residues in this structure from the 88 obtained using *phenix.resolve* sequence assignment alone (Terwilliger, 2003 ▶) to 247 of a possible 359. Additionally, the procedure was tested by application to a series of templates with sequence identities to a target structure ranging between 7 and 36%. The mean fraction of correctly identified residues in these cases was increased from 33% using *phenix.resolve* sequence assignment to 47% using the current procedure. The procedure is simple to apply and is available in the *Phenix* software package.

## Abstract 2

A procedure for model building is described that combines morphing a model to match a density map, trimming the morphed model and aligning the model to a sequence.

## 1. Introduction

Molecular replacement (Rossmann, 1972 ▶) is an exceptionally powerful method for macromolecular structure determination and is now used in obtaining most new macromolecular structures. A key step in molecular replacement is the use of the newly placed working model to calculate crystallographic phases and an electron-density map that can be used to build a new and more accurate model. If the working model is very different from the true structure (more than about 1.5–2.0 Å r.m.s.d. over main-chain atoms) then this step can be difficult as the calculated phases may be inaccurate. To overcome this difficulty, methods have been developed to modify the model after it has been placed in the crystallographic cell, making it more similar to the true structure. These methods range greatly in complexity from rigid-body or full refinement of the model to simulated-annealing refinement (Brünger *et al.*, 1998 ▶), DEN (Schröder *et al.*, 2010 ▶), jelly-body refinement (Murshudov *et al.*, 2011 ▶), iterative model rebuilding and refinement (Perrakis *et al.*, 1999 ▶; Cohen *et al.*, 2008 ▶; Cowtan, 2006 ▶; Terwilliger *et al.*, 2008 ▶), and combination of crystallo­graphic refinement with *Rosetta* structure-modeling tools (DiMaio *et al.*, 2011 ▶; Terwilliger, DiMaio *et al.*, 2012 ▶).

We recently developed an additional procedure that we term ‘morphing’ for improving a model after it has been placed by molecular replacement (Terwilliger, Read *et al.*, 2012 ▶). Morphing can be thought of as an automated way to apply a smooth deformation to a model to make it match an electron-density map more closely. Morphing is useful in the common situation in which the model is locally similar to the true structure but there are many small differences in dihedral angles along the chain so that parts of the model and the true structure that are separated by many residues cannot be superimposed closely.

**Figure 1:**
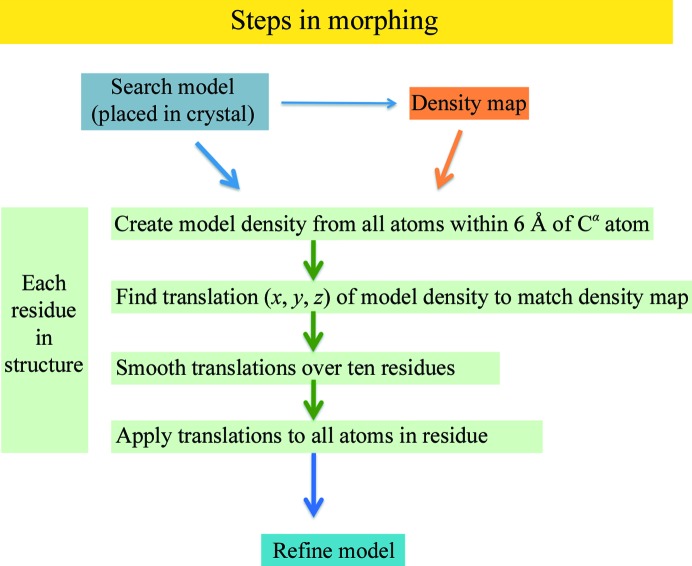
Schematic of morphing.

Fig. 1 ▶ illustrates the steps in morphing. The overall goal is to deform a structure in a smooth way that leads to a better fit to a density map and that at the same time leads to a model that is closer to the target structure. The basic idea is to find local translations that can be applied to each small part of the model and improve their fit to the density. After applying all of these translations, the resulting model may be improved as well. Firstly, local translations are identified for each residue in the structure. All of the atoms in the current model within a radius of 6 Å of a particular C^α^ atom are considered. Model density is calculated from these atoms. The correlation (fit) of the model density and the density in the map are compared, and a small translation (typically up to 2 Å) of the model density is found that maximizes this correlation. Once these translations (vector shifts) have been identified for all of the residues in a segment of structure, they are smoothed, typically in a window of ten residues. Finally, the smoothed shifts are applied to all of the atoms of the corresponding residue. This procedure maintains the geometry of individual residues, but can result in poor geometry for the connecting residues. The morphed model is then refined to improve the geometry. The entire procedure can then be iterated by generating a new electron-density map and morphing again.

As described recently (Terwilliger, Read *et al.*, 2012 ▶), the convergence of structures towards the correct model using morphing is intermediate between that obtained with refinement alone and that obtained with the more powerful combination of *Rosetta* structure modeling with crystallo­graphic refinement and model building (*phenix.mr_rosetta*; DiMaio *et al.*, 2011 ▶; Terwilliger, DiMaio *et al.*, 2012 ▶). The effectiveness of morphing can also be increased by combin­ation with DEN refinement (Brunger *et al.*, 2012 ▶). The computation required to carry out morphing is similar to that required for extensive refinement. For a representative set of structures (DiMaio *et al.*, 2011 ▶; Terwilliger, Read *et al.*, 2012 ▶), the time required to carry out extensive refinement (100 cycles) was from 1 to 5.5 h; morphing took from 1 to 5 h and *phenix.mr_rosetta* took from 30 to 130 h. Morphing can be applied in addition to standard procedures used in macromolecular crystallography such as density modification and automated model building. As morphing distorts the template to be a little more like the target protein, it can provide a somewhat more effective starting point for these standard procedures. The electron-density map used in morphing can be any map, but typically a prime-and-switch density-modified map is used (Terwilliger, 2004 ▶). Morphing has a significant limitation in that it does not change the residues in the chain, only their coordinates. Consequently, if the true structure has fewer or more residues, or different residues, than the working model, these differences cannot be corrected by morphing.

Here, we describe a procedure that helps to address this limitation in morphing. The goal of the procedure is to obtain a relatively complete model of the structure that has accurate main-chain atomic positions and residues that are in general correctly assigned to sequence. After carrying out morphing, the morphed model is iteratively rebuilt using automated model building, density modification and refinement (*e.g.* with *phenix.autobuild*; Terwilliger *et al.*, 2008 ▶) in order to obtain a high-quality density-modified map. This density map is then used along with the original morphed model in the following steps. Firstly, all of the residues in the morphed model that do not match the electron-density map are removed. The sequence assignment of each segment of the resulting trimmed morphed model is then identified using the density in the map, the connectivity of the chains in the template model and loops that can be found from the electron-density map. Once the sequence assignment has been identified, any loops that are consistent with this assignment are used to connect segments together. The procedure can be iterated to improve the sequence assignment and completeness.

## 2. Methods

### 2.1. Morphing of placed model

Morphing of a placed model was carried out as described previously (Terwilliger, Read *et al.*, 2012 ▶). The starting template (placed model) can be used to calculate a prime-and-switch electron-density map (Terwilliger, 2004 ▶). A distortion that varies smoothly along the chain is then applied to the model to optimize its fit to the electron-density map. The resulting model is refined and the procedure is repeated six times, yielding a morphed model.

### 2.2. Calculation of a density-modified map after iterative model rebuilding

An optimized electron-density map is calculated starting from the morphed model by iterative model rebuilding and density modification. The morphed model is automatically rebuilt by *phenix.autobuild* (Terwilliger *et al.*, 2008 ▶) using default parameters, except that the full rebuilding mode (the rebuild_in_place=False option on the command line, for example) is used so that the model will be fully rebuilt. The final density-modified electron-density map from this rebuilding procedure is used in subsequent procedures that require an electron-density map. The model itself could, in principle, be used in subsequent steps; however, in the method described here it is not used. This is because the autobuilt model might no longer have the full connectivity present in the starting model and the method (see below) benefits substantially from having this connectivity information.

### 2.3. Trimming the morphed model

The morphed model is trimmed by removing residues that poorly match the density-modified electron-density map obtained above. This procedure can be carried out with *phenix.autobuild* (Terwilliger *et al.*, 2008 ▶). The criterion for the removal of residues is based on the average density in the map at the positions of atoms in the morphed model. Residues are rejected if the mean density for atoms in that residue (ρ_mean_) is low compared with that of all residues (ρ_mean_ < 0.5ρ_mean_all_). An additional optional term further removes some additional residues that might be below this threshold if there were no noise in the map (by default, those within 0.2 times SD_mean_all_, the standard deviation of the values of ρ_mean_all_). This leads to removal of residues with ρ_mean_ < 0.5ρ_mean_all_ + 0.2SD_mean_all_. Additionally, all residues for which the local correlation between the map and the density calculated from the model is less than 0.4 are rejected. These criteria were obtained by optimizing the match of the residues remaining after trimming with the known structure of Cgl1109 in the test case described below. They are adjustable and it may be useful to try several values for any particular case, visually examining which residues have been removed and their fit to density.

### 2.4. Sources of information for sequence assignment of the trimmed morphed model

The assignment of each residue in a model to a residue in the sequence of the target structure is the core step in this procedure. The goal is to identify the residue types associated with as many residues in the model as possible, while having as few incorrectly assigned as possible.

Some sources of information for sequence assignment are independent of the template. These include a probabilistic assignment for each segment in the model based on electron density in the map (see, for example, Terwilliger, 2003 ▶) and constraints based on the distances between ends of segments. Additional information that is independent of the template comes from estimates of the lengths of connections between segments based on loops fitting the electron density that can be built connecting them. Finally, an important constraint is that each part of the sequence of the target molecule can only be used once (or, if noncrystallographic symmetry is present, up to the number of copies).

Further information on sequence assignment is available if the model is based on a template with a known sequence, such as is typically the case in molecular replacement. In this case it is likely that the sequence of the template and the sequence of the target structure are related. Therefore, a sequence alignment relating the template and the target structure contains information about the desired sequence for the morphed model. A final important source of information is that the order of the segments in the target structure is likely to be the same as the order of these segments in the template structure. This constraint corresponds to assuming that the two structures differ only in coordinates, insertions and deletions, not in swapping of the order of segments.

### 2.5. Procedure for sequence assignment of the trimmed morphed model

Sequence assignment is carried out by listing all of the possible assignments for each segment in the current working model and then finding a set of sequence assignments (one for each segment) that optimizes a target function based on the criteria described in the preceding section. This search is carried out by first finding the segment that has the most well defined position based on side-chain density in the electron-density map and then iteratively trying all possible additions of additional segments, picking the highest-scoring assignment or assignments at each stage. In cases where a template structure is available with a connectivity that is anticipated to be the same as the target structure, the total number of possibilities is limited and a more complete search can be carried out in which most or all placements of all segments are tested.

### 2.6. A scoring function for sequence assignment of the trimmed morphed model

The procedure described above for sequence assignment requires a target function that can be optimized. The target function is calculated from the assignment of one or more segments to the sequence. Terms in the target function are additive. The default values of all adjustable parameters were set by optimizing the number of residues that are correctly assigned in the test case Cgl1109 described below. In practice, the process was found not to be very sensitive to the exact values of these parameters; however, the software allows the values to be modified if desired.

**Figure 2:**
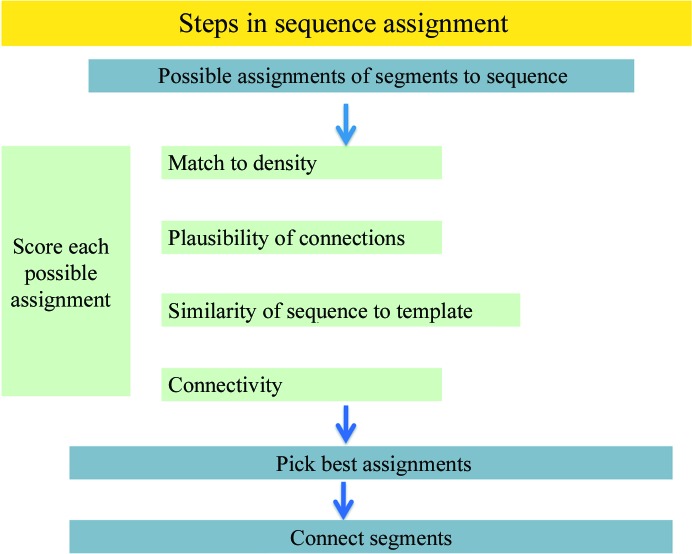
Schematic of sequence assignment.

The principal terms in the target function are the match of the side chains to the density, the plausibility of connections between segments, the changes in sequence from the template and the connectivity of the model (Fig. 2 ▶). The score for the match of the side chains to the density is the logarithm of the estimated probability of a correct assignment (as described in Terwilliger, 2003 ▶), so an assignment that has an estimated 5% chance of being correct would receive a score 2.95 units lower than one with an estimated 95% chance of being correct. The plausibility of a connection is based on the number of residues between the ends of adjacent segments and the distance between these ends. (If the distance is greater than can possibly be spanned by this number of peptides, then the assignment is not possible and is rejected). If instead a loop matching the density map can be built, then the assignment receives a favourable additional term (typically ten units). An optional term reflects differences between the sequence assignment and the original sequence of a segment. The value of this term is a *Z*-score for agreement between these sequences (the number of standard deviations of the alignment score above the mean for all possible alignments) and is zero if the *Z*-score is negative, where the alignment score is based on the BLOSUM62 scoring matrix (Henikoff & Henikoff, 1992 ▶). If there is no noncrystallographic symmetry, and two segments in the trimmed model are assigned to the same or overlapping residues in the sequence, then the assignment is again rejected. If the template used to begin the process is assumed to have the same connectivity as the final structure, then any assignment that changes the order of any segment is also rejected.

### 2.7. Iteration of sequence assignment allowing additional loops to be tested

The scoring and optimization procedure described above includes favorable terms for pairs of segments that (i) can be connected by a loop containing the appropriate number of residues and (ii) match the density map. It is impractical to test all possible loops between all segments, so instead loops are built between ends of segments that are close together and between ends of segments that are assigned to parts of the sequence that are separated by only a few residues. At the beginning of the procedure, few or none of the segments of the trimmed morphed model may be assigned to sequence, so this second approach may not be applicable. After carrying out optimization, one or more high-scoring sequence assignments may be obtained. A new model is then constructed based on the highest-scoring sequence assignment. This new model may have new loops connecting segments in the previous model, creating new longer segments. This new model is then used as the basis for a new optimization and a new set of attempts to build loops.

## 3. Results and discussion

### 3.1. Test case at a resolution of 3.2 Å

To develop and assess the effectiveness of our procedure for sequence assignment, we chose a challenging molecular-replacement structure solution that we recently completed using a combination of DEN refinement, automated and manual procedures (Brunger *et al.*, 2012 ▶). The structure to be determined was Cgl1109 (Joint Center for Structural Genomics target 376512 listed in TargetTrack; http://targetdb.sbkb.org/tt), a putative succinyl-diaminopimelate desuccinylase from *Corynebacterium glutamicum*. The data for this structure are highly anisotropic and the data set used in this work extended to a resolution of 3.2 Å. The template used as a starting point for this structure determination was the structure of a succinyl-diaminopimelate desuccinylase from the β-proteobacterium *Neisseria meningitidis* (PDB entry 1vgy; Badger *et al.*, 2005 ▶; Berman *et al.*, 2000 ▶) that had been edited and placed in the correct position in the unit cell of the structure to be determined (DiMaio *et al.*, 2011 ▶). The r.m.s.d. (main-chain atoms) between this starting model and the target structure was 2.5 Å and the sequence identity was 20%.

### 3.2. Morphing, obtaining a density-modified map after autobuilding and trimming

**Figure 3:**
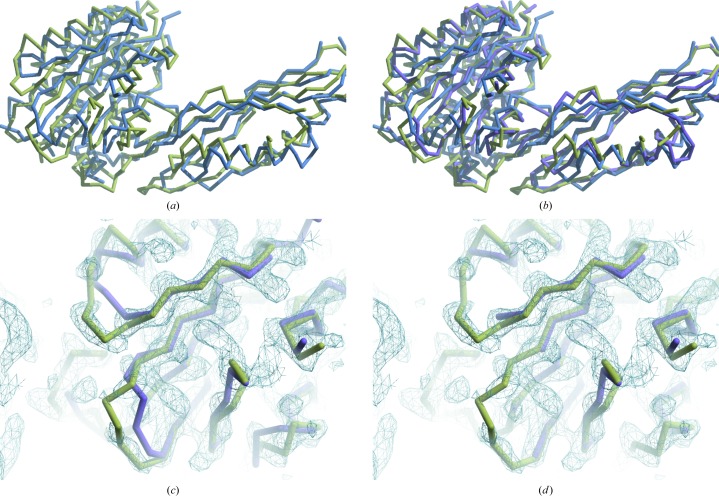
(*a*) Template structure 1vgy (Badger *et al.*, 2005 ▶; blue) superimposed on the final model of Cgl1109 (Brunger *et al.*, 2012 ▶; yellow). (*b*) As in (*a*) but with the morphed model of 1vgy (purple). (*c*) Morphed model (purple) based on 1vgy, the final structure (yellow) and the *phenix.autobuild* density-modified electron-density map obtained from the morphed model. The map was corrected for anisotropy and sharpening to an effective *B* factor of 32 Å^2^. (*d*) Trimmed morphed model (purple) obtained from the map and model in (*c*); the structure of Cgl1109 is shown in yellow. All maps are contoured at 1.5σ and all figures were prepared with *Coot* (Emsley *et al.*, 2010 ▶).

Morphing of the desuccinylase template based on the data for the Cgl1109 structure has been described previously (Terwilliger, Read *et al.*, 2012 ▶). Fig. 3 ▶(*a*) shows the template structure 1vgy (blue) superimposed on the final model of Cgl1109 (yellow). Fig. 3 ▶(*b*) adds the morphed model (purple). It can be seen that morphing moves the 1vgy structure closer to the final model (the r.m.s.d. of the main-chain atoms after morphing was 1.8 Å, reduced from 2.5 Å for the template; Terwilliger, Read *et al.*, 2012 ▶).

The morphed model was then used as a starting point for creating an improved electron-density map by carrying out automated model building, density modification and refinement with *phenix.autobuild* (Terwilliger *et al.*, 2008 ▶). Fig. 3 ▶(*c*) shows a portion of the morphed model (purple) and this density-modified electron-density map, along with the final structure of Cgl1109 for comparison (yellow). The map obtained from this process was considerably improved over the map used in morphing, although the autobuilding process resulted in a model that was incompletely assigned to sequence (167 residues of a possible 359 assigned to sequence; *R* and *R*_free_ of 0.32 and 0.38, respectively). Fig. 3 ▶(*d*) shows the trimmed model (purple) obtained by removing residues from the morphed model in Fig. 3 ▶(*c*) that poorly match the density in the density-modified map in Fig. 3 ▶(*c*). It can be seen from Fig. 3 ▶(*d*) that trimming to remove residues that do not match the density map effectively removes residues that do not match the final structure of Cgl1109.

### 3.3. Sequence assignment of the morphed trimmed model

**Figure 4:**
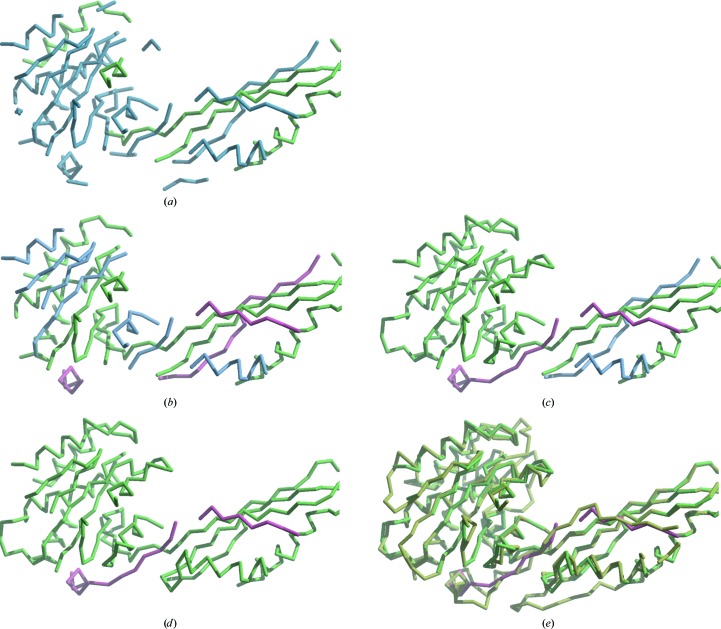
(*a*) The morphed trimmed model as shown in Fig. 3 ▶(*d*) is colored to indicate the segments that were correctly assigned to sequence (green) and those that were not assigned to sequence (blue) using *phenix.resolve* sequence assignment (Terwilliger, 2003 ▶). (*b*) Sequence assignment and loop building for the morphed trimmed model using *phenix.assign_sequence*, including information on loops that can be built connecting ends of the model and not allowing overlapping sequences. Coloring is as in (*a*), with segments that are incorrectly assigned in red. (*c*) Sequence assignment as in (*b*), requiring that the order of the segments in the model match that in the template. (*d*) As in (*c*), but iterating the assignment process. (*e*) As in (*d*), but superimposed on the final model of Cgl1109 (yellow).

We used the map in Fig. 3 ▶(*d*) as a reference for assigning sequence to the trimmed model in Fig. 3 ▶(*d*). To illustrate the roles of each of the most important sources of information in sequence assignment, we sequentially applied selected sources of information in the sequence assignment of the morphed trimmed model. Fig. 4 ▶(*a*) shows sequence assignment including only probabilistic matching to density. The morphed trimmed model (as in Fig. 3 ▶ *d*) is colored in Fig. 4 ▶(*a*) to indicate the segments that were correctly assigned to sequence (green) and those that were not assigned to sequence (blue). In this case 240 of the 359 residues in the structure were built and 88 residues were assigned to sequence (all 88 were correctly assigned). Fig. 4 ▶(*b*) shows sequence assignment as in Fig. 4 ▶(*a*) but also including constraints on non-overlapping sequences and optimization for consistency with loops that can be built. Residues assigned to sequence incorrectly are indicated in red. With this additional information, a total of 151 residues of the 273 built were correctly assigned to sequence. Fig. 4 ▶(*c*) shows sequence assignment as in Fig. 4 ▶(*b*), but also requiring that the order of the segments in the model be the same as the order in the template. With this information, a total of 218 of the 270 residues built were correctly assigned. Fig. 4 ▶(*d*) shows sequence assignment as in Fig. 4 ▶(*c*), except that the procedure was iterated three times, allowing additional loop information to be generated. This yielded a total of 247 of the 276 residues built correctly assigned to sequence. Fig. 4 ▶(*e*) shows the model in Fig. 4 ▶(*e*) superimposed on the final model of Cgl1109 (yellow). It may be seen that the residues in the model that are incorrectly assigned to sequence (shown in red) are poor matches to the final model, while the remaining residues in the model generally match the final model well.

### 3.4. Application to morphing and sequence assignment of a series of templates

**Figure 5:**
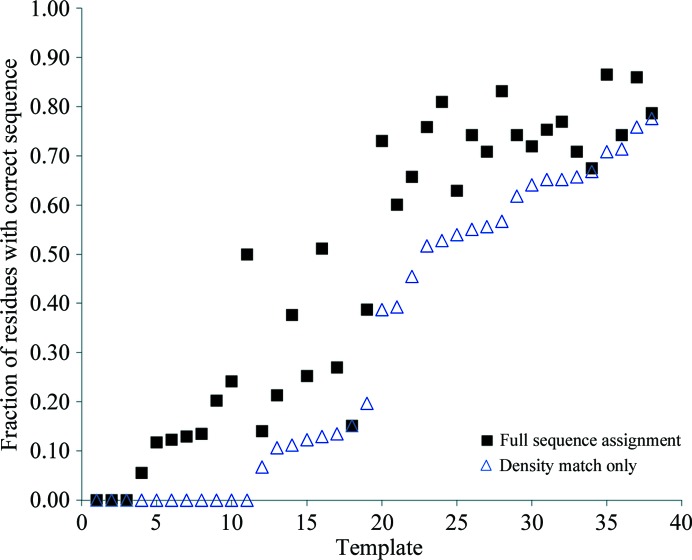
Sequence assignment of a series of templates with and without inclusion of information on connectivity of the template (see text for details). The fraction of residues assigned correctly for the structure 1a2b obtained using each template is shown. The open triangles reflect assignments using only *phenix.resolve* sequence assignment based on the match of the side-chain density to the map and the filled squares reflect assignments including the full procedure described here.

The approach described above was further tested by applying it to a series of starting models with varying simil­arities to a target structure. The structure to be determined was that of human RhoA (PDB entry 1a2b; Ihara *et al.*, 1998 ▶; the GTP analogue in the crystal was ignored in this test). A set of 38 structures similar in sequence to 1a2b were identified by sequence alignment using *HHpred* (Söding, 2005 ▶) and were placed in the same location in the unit cell as 1a2b (DiMaio *et al.*, 2011 ▶). These structures range from 7 to 36% sequence identity to 1a2b. Each of these structures in turn was used as a template for morphing based on the structure factors for 1a2b, followed by autobuilding to obtain an improved map, trimming to match the new map and sequence assignment. All calculations were carried out with the default parameters obtained from the analysis of the Cgl1109 structure above. Fig. 5 ▶ shows the fraction of residues correctly assigned for each template either using only matching of side-chain density to the map for sequence assignment (open triangles) or the full procedure described here. The templates are sorted according to the fraction of residues that were correctly assigned using only matching of side-chain density. It may be seen from Fig. 5 ▶ that the full procedure improves the accuracy of the sequence-assignment process for nearly all of the templates examined. The mean fraction of residues that were correctly assigned using only *phenix.resolve* matching of side-chain density was 33%. Using the full procedure the mean fraction correctly assigned increased to 47%.

## 4. Conclusions

We find that a combination of morphing, trimming and sequence assignment including information from a template structure and requiring non-overlapping assignments of segments can be of substantial utility in the assignment of sequence to a model obtained by molecular replacement. Although we have developed and demonstrated our approach for morphing and sequence assignment using *Phenix* tools for model building and probabilistic sequence assignment, the general approaches could be carried out with any model-building and sequence-assignment methods. We would expect in particular that adding information on sequence assignment based on non-overlap and on the order of segments in the template could substantially improve sequence assignment in any methods that do not already use this information.

## 5. Availability

The *phenix.morph_model* and *phenix.assign_sequence* tools that can carry out morphing and sequence assignment and instructions for their use are available as part of the *Phenix* GUI (http://www.phenix-online.org; Echols *et al.*, 2012 ▶), which is freely available to academic users both as binaries for standard Macintosh, Windows and Linux platforms, and as source code.
